# Optimization of Molecules via Deep Reinforcement Learning

**DOI:** 10.1038/s41598-019-47148-x

**Published:** 2019-07-24

**Authors:** Zhenpeng Zhou, Steven Kearnes, Li Li, Richard N. Zare, Patrick Riley

**Affiliations:** 1 Department of Chemistry, Stanford University, Stanford, California USA; 2 Google Research Applied Science, Mountain View, California USA; 3 Work done during an internship at Google Research Applied Science, Mountain View, California USA

**Keywords:** Cheminformatics, Cheminformatics

## Abstract

We present a framework, which we call Molecule Deep *Q*-Networks (MolDQN), for molecule optimization by combining domain knowledge of chemistry and state-of-the-art reinforcement learning techniques (double *Q*-learning and randomized value functions). We directly define modifications on molecules, thereby ensuring 100% chemical validity. Further, we operate without pre-training on any dataset to avoid possible bias from the choice of that set. MolDQN achieves comparable or better performance against several other recently published algorithms for benchmark molecular optimization tasks. However, we also argue that many of these tasks are not representative of real optimization problems in drug discovery. Inspired by problems faced during medicinal chemistry lead optimization, we extend our model with multi-objective reinforcement learning, which maximizes drug-likeness while maintaining similarity to the original molecule. We further show the path through chemical space to achieve optimization for a molecule to understand how the model works.

## Introduction

One fundamental goal in chemistry is to design new molecules with specific desired properties. This is especially important in material design or drug screening. Currently, this process is expensive in terms of time and cost: It can take years and cost millions of dollars to find a new drug^1^. The goal of this study is to partially automate this process through reinforcement learning.

To appreciate our approach, it is necessary to review briefly the previous works that employed machine learning in molecule design. One prevalent strategy is to build a generative model, which maps a point in a high-dimensional latent space to a molecule, and perform search or optimization in the latent space to find new molecules. Gómez-Bombarelli *et al*.^2^, Blaschke *et al*.^3^, Segler *et al*.^4^, Lim *et al*.^5^, and Putin *et al*.^6^ utilized strings as molecule representations to build a generator of SMILES^7^ strings, which is a linear string notation to describe molecular structures. One of the most challenging goals in this design is to ensure the chemical validity of the generated molecules. Kusner *et al*.^8^ and Dai *et al*.^9^ added grammar constraints to SMILES strings to improve the chemical validity of the generated molecules. Researchers have also built models on graph representations of molecules, which regards atoms as nodes and bonds as edges in an undirected graph. Li *et al*.^10^ and Li *et al*.^11^ described molecule generators that create graphs in a step-wise manner. De Cao & Kipf^12^ introduced MolGAN for generating small molecular graphs. Jin *et al*.^13^ designed a two-step generation process in which a tree is first constructed to represent the molecular scaffold and then expanded to a molecule. Although almost perfect on generating valid molecules, these autoencoder-based models usually need to address the problem of optimization. Most published work uses a separate Gaussian process model on the latent space for optimization. However, because the latent space is often high dimensional and the objective functions defined on the latent space is usually non-convex, molecule property optimization on the latent space can be difficult.

Another strategy is based on reinforcement learning, which is a sub-field of artificial intelligence. Reinforcement learning studies the way to make decisions to achieve the highest reward. Olivecrona *et al*.^14^, Guimaraes *et al*.^15^, Putin *et al*.^16^, and Popova *et al*.^17^ applied reinforcement learning techniques on top of a string generator to generate the SMILES strings of molecules. They successfully generated molecules with given desirable properties, but struggled with chemical validity. Recently, You *et al*.^18^ proposed a graph convolutional policy network (GCPN) for generating graph representations of molecules with deep reinforcement learning, achieving 100% validity. However, all these methods require pre-training on a specific dataset. While pre-training makes it easier to generate molecules similar to the given training set, the exploration ability is limited by the biases present in the training data.

Here we introduce a new design for molecule optimization by combining chemistry domain knowledge and reinforcement learning, which we call Molecule Deep *Q*-Networks (MolDQN). We formulate the modification of a molecule as a Markov decision process (MDP)^19^. By only allowing chemically valid actions, we ensure that all the molecules generated are valid. We then employ the deep reinforcement learning technique of Deep *Q*-Networks (DQN)^20^ to solve this MDP, using the desired properties as rewards. Instead of pre-training on a dataset, our model learns from scratch. Additionally, with the introduction of multi-objective deep reinforcement learning, our model is capable of performing multi-objective optimization.

Our contribution differs from previous work in three critical aspects:

1. All the works presented above use policy gradient methods, while ours is based on value function learning. Although policy gradient methods are applicable to a wider range of problems, they suffer from high variance when estimating the gradient^21^. In comparison, in applications where value function learning works, it is usually more stable and sample efficient^20^.
2. Most, if not all, of the current algorithms rely on pre-training on some datasets. Although expert pre-training may lead to lower variance, this approach limits the search space and may miss the molecules which are not in the dataset. In contrast, our method starts from scratch and learns from its own experience, which can lead to better performance, i.e., discovering molecules with better properties.
3. Our model is designed for multi-objective reinforcement learning, allowing users to decide the relative importance of each objective. See 3.3 for more detail.

## Methods

### Molecule modification as a markov decision process

Intuitively, the modification or optimization of a molecule can be done in a step-wise fashion, where each step belongs to one of the following three categories: (1) atom addition, (2) bond addition, and (3) bond removal. The molecule generated is only dependent on the molecule being changed and the modification made. Therefore, the process of molecule optimization can be formulated as a Markov decision process (MDP). We have several key differences from previous work that employed MDP for molecule modification^18^.

**Figure 1:**
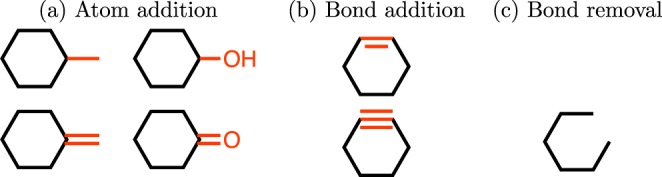
Valid actions on the state of cyclohexane. Modifications are shown in red. Invalid bond additions which violate the heuristics explained in Section 2.1 are not shown.
  2. Bond addition. A bond addition action is performed between two atoms with free valence (not counting implicit hydrogens). If there is no bond between those two atoms, actions between them consist of adding a single, double, or triple bond if the valence allows this change. Additional actions *increase* the bond order between those two atoms by one or two. In other words, the transitions include:
    No bond → {Single, Double, Triple} Bond.
    Single bond → {Double, Triple} Bond.
    Double bond → {Triple} Bond.
    To generate molecules that are chemically more reasonable, we include several heuristics that incorporate chemistry domain knowledge. First, in order to prevent generating molecules with high strain, we do not allow bond formation between atoms that are in rings. In addition, we added an option that only allows formation of rings with a specific number of atoms. Note that it is possible to get a 7-membered ring, even when only rings with 3–6 atoms are allowed, by creating a bicyclic structure and then removing the bridging bond (Fig. S8). As an example, Fig. 1b shows the allowed bond addition actions for cyclohexane.
  3. Bond removal. We define the valid bond removal action set as the actions that decrease the bond order of an existing bond. The transitions include:
    Triple bond → {Double, Single, No} Bond.
    Double bond → {Single, No} Bond.
    Single bond → {No} Bond.
    Note that bonds are only completely removed if the resulting molecule has zero or one disconnected atom (and in the latter case, the disconnected atom is removed as well). Therefore, no molecules having disconnected parts are created in this step.

- We add an explicit limit on the number of steps. This allows us to easily control how far away from a starting molecule we can go. In vast chemical space, this is a very natural way to control the diversity of molecules produced.
- We do not allow chemically invalid actions (violations of valence constraints). These actions are removed from the action space entirely and are not even considered by our model.
- We allow atoms/bonds to be removed as well as added.
  Formally, we have MDP$({\mathscr{S}},{\mathscr{A}},\{{P}_{sa}\}, {\mathcal R} )$, where we define each term in what follows:
- ${\mathscr{S}}$ denotes the state space, in which each state $s\in {\mathscr{S}}$ is a tuple of $(m,t)$. Here *m* is a valid molecule and *t* is the number of steps taken. For the initial state, the molecule *m* can be a specific molecule or nothing, and $t=0$. We limit the maximum number of steps *T* that can be taken in this MDP. In other words, the set of terminal states is defined as $\{s=(m,t)|t=T\}$, which consists of the states whose step number reaches its maximum value.
- ${\mathscr{A}}$ denotes the action space, in which each action $a\in {\mathscr{A}}$ is a valid modification to a specific molecule *m*. Each modification belongs to one of the following three categories mentioned before:
  1. Atom addition. Firstly, we define the set of ${\mathcal E}$ be the set of elements a molecule contains. We then define a valid action as adding (1) an atom in ${\mathcal E}$ and (2) a bond between the added atom and the original molecule wherever possible (all valence-allowed bond orders are considered as separate actions). For example, with the set of elements ${\mathcal E} =\{{\rm{C}},{\rm{O}}\}$, the atom addition action set of cyclohexane contains the 4 actions shown in Fig. 1a. Note that hydrogens are considered implicitly, and all atom additions are defined as replacements of implicit hydrogens.

In our design choice, we do not break an aromatic bond. However, it is still possible to break aromaticity. (See the third molecule generated in Section 3.3, $w=0.4$; the removal of the extracyclic double bond from the original molecule breaks aromaticity.) Besides, an aromatic system can still be created in a stepwise way by adding single and double bonds alternatively, and the resulting system will be perceived as aromatic by the RDKit SMILES parser. We also include “no modification” as an action, which allows the molecule to remain unchanged before reaching the step limitation *T*.

- {*P*_*sa*_} denotes the state transition probability. Here we define the state transition to be deterministic. For example, if we modify a molecule by adding a single bond, the next state we reach will be the new molecule adding the bond, with a probability of 1.
- ${\mathcal R}$ denotes the reward function of state $(m,t)$. In material design or lead optimization, the reward is often a property of the molecule *m*. In our design, a reward is given not just at the terminal states, but at each step, which empirically produces better learning performance (see Fig. S3). To ensure that the final state is rewarded most heavily, we discount the value of the rewards at a state with time *t* by *γ*^*T*–*t*^ (where we typically used *γ* = 0.9). Note that the definition of discount factor is different from the usual way. In future discussions of reward *r*_*t*_, this discount factor is implicitly included for simplicity.

#### Implementation details

We implemented the state transition of a molecule with the available software framework of RDKit^22^. The properties of molecules are calculated with tools provided by RDKit.

### Reinforcement Learning

Reinforcement Learning is an area of machine learning concerning how the *decision makers* (or *agents*) ought to take a series of actions in a prescribed *environment* so as to maximize a notion of cumulative reward, especially when a model of the environment is not available. Here, the environment is the molecule modification MDP we defined above, and our goal is to find a policy *π* which selects an action for each state that can maximize the future rewards.

Intuitively, we are trying to fit a function $Q(s,a)$ that predicts the future rewards of taking an action *a* on state *s*. A decision is made by choosing the action *a* that maximizes the *Q* function, which leads to larger future rewards.

Mathematically, for a policy *π*, we can define the value of an action *a* on a state *s* to be

$${Q}^{\pi }(s,a)={Q}^{\pi }(m,t,a)={{\mathbb{E}}}_{\pi }[\sum _{n=t}^{T}\,{r}_{n}]$$

where ${{\mathbb{E}}}_{\pi }$ denotes taking an expectation with respect to *π*, and *r*_*n*_ denotes the reward at step *n*. This action-value function calculates the future rewards of taking action *a* on state *s*, and subsequent actions decided by policy *π*. We can therefore define the optimal policy ${\pi }^{\ast }(s)={\rm{\arg }}\,{{\rm{\max }}}_{a}{Q}^{{\pi }^{\ast }}(s,a)$.

In our case, however, we have both a deterministic MDP and an accurate model of the environment. Therefore, we chose to approximate the value function $V(s)={{\rm{\max }}}_{a}\,Q(s,a)$ and we calculate the *Q* function for an action *a* moving from state *s* to *s*′ as $Q(s,a)= {\mathcal R} (s^{\prime} )+V(s^{\prime} )$

Under the setting that the maximum number of steps is limited, the MDP is time-dependent, and the optimal policy will be time-dependent as well. Naturally, if there are many steps left, we can risk pursuing later but larger rewards, while if only a few steps remain, we should focus on rewards that can be obtained sooner.

We adopt a deep *Q*-learning^20^ algorithm to find an estimate of the *Q* function. We refer to a neural network function approximator as the parameterized *Q*-value function $Q(s,a;\theta )$, where *θ* is the parameter. This approximator can be trained by minimizing the loss function of

$$l(\theta )={\mathbb{E}}[{f}_{l}({y}_{t}-Q({s}_{t},{a}_{t};\theta ))]$$

where ${y}_{t}={r}_{t}+{{\rm{\max }}}_{a}\,Q({s}_{t+1},a;\theta )$ is the target value, and *f*_*l*_ is a loss function. In our case, we use the Huber loss^23^ as a loss function.

$${f}_{l}(x)=\{\begin{array}{ll}\frac{1}{2}{x}^{2} & {\rm{if}}\,|x| < 1\\ |x|-\frac{1}{2} & {\rm{otherwise}}\end{array}$$

### Multi-objective reinforcement learning

In real-world applications like lead optimization, it is often desired to optimize several different properties at the same time. For example, we may want to optimize the selectivity of a drug while keeping the solubility in a specific range. Formally, under the multi-objective reinforcement learning setting, the environment will return a vector of rewards at each step *t*, with one reward for each objective, i.e. ${\overrightarrow{r}}_{t}={[{r}_{1,t},\ldots ,{r}_{k,t}]}^{T}\in {{\mathbb{R}}}^{k}$, where *k* is the number of objectives.

There exist various goals in multi-objective optimization. The goal may be finding a set of Pareto optimal solutions, or find a single or several solutions that satisfy the preference of a decision maker. Similar to the choice in Guimaraes *et al*.^15^, we adapted the latter one in this paper. Specifically, we implemented the “scalarized” reward framework to realize multi-objective optimization, with the introduction of a user defined weight vector $w={[{w}_{1},{w}_{2}\ldots ,{w}_{k}]}^{T}\in {{\mathbb{R}}}^{k}$, the scalarized reward can be calculated as

$${r}_{s,t}={w}^{T}{\overrightarrow{r}}_{t}=\sum _{i=1}^{k}\,{w}_{i}{r}_{i,t}$$

The objective of the MDP is then to maximize the cumulative scalarized reward.

### Exploitation vs. exploration during training

The trade-off between exploitation and exploration presents a dilemma caused by the uncertainty we face. Given that we do not have a complete knowledge of the rewards for all the states, if we constantly choose the best action that is known to produce the highest reward (*exploitation*), we will never learn anything about the rewards of the other states. On the other hand, if we always chose an action at random (*exploration*), we would not receive as much reward as we could achieve by choosing the best action.

One of the simplest and the most widely used approaches to balance these competing goals is called $\varepsilon$-greedy, which selects the predicted best action with probability $1-\varepsilon$, and a uniformly random action with probability $\varepsilon$. Without considering the level of uncertainty of the value function estimate, $\varepsilon$-greedy often wastes exploratory effort on the states that are known to be inferior.

To counter this issue, we followed the idea of bootstrapped-DQN from Osband *et al*.^24^ by utilizing randomized value functions to achieve deep exploration. We built *H* independent *Q*-functions $\{{Q}^{(i)}|i=1,\ldots ,H\}$ (actually, a multi-task neural network with a separate head for each ${Q}^{(i)}$; see Section 2.5), each of them being trained on a different subset of the samples. At each episode, we uniformly choose $i\in \{1,\ldots ,H\}$, and use ${Q}^{(i)}$ for decision making. The above approach is combined with $\varepsilon$-greedy as our policy. During training, we annealed $\varepsilon$ from 1 to 0.01 in a piecewise linear way.

### Deep *Q*-learning implementation details

We implemented the deep *Q*-learning model described by Mnih *et al*.^20^ with improvements of double *Q*-learning^25^. Recall that a state *s* is a pair of molecule *m* and time *t*. Unsurprisingly, including *t* in the model performs better experimentally (see Fig. S4).

We used a deep neural network to approximate the *Q*-function. The input molecule is converted to a vector form called its Morgan fingerprint^26^ with radius of 3 and length of 2048, and the number of steps remaining in the episode was concatenated to the vector. A four-layer fully-connected network with hidden sizes of [1024, 512, 128, 32] and ReLU activations is used as the network architecture. Its output dimension is the number *H* (see above; for computational efficiency, we implemented these *H* different models as multiple outputs on top of shared network layers). In the single property optimization task, we only allow generation with ring sizes of 5, 6, and 7; while in all other experiments, we allow ring sizes of 5 and 6. In most experiments, we limited the maximum number of steps per episode to 40, given that most drug molecules have less than 40 atoms (the exception is for the experiments in Section 3.1, where we limit the max number of steps to be 38 for logP optimization to match You *et al*.^18^, and Section 3.2, where the limit is 20.). We trained the model for 5,000 episodes with the Adam optimizer^27^ with a learning rate of 0.0001. We used $\varepsilon$-greedy together with randomized value functions as a exploration policy, and, as mentioned before, we annealed $\varepsilon$ from 1 to 0.01 in a piecewise linear way. The discount factor *γ* (as defined in Section 2.1) was set to 0.9.

## Results and Discussion

In these tasks, we demonstrated the effectiveness of our framework on optimizing a molecule to achieve desired properties. We compared MolDQN with the following state-of-the-art models:

- Junction Tree Variational Autoencoder (JT-VAE)^13^ is a deep generative model that maps molecules to a high-dimensional latent space and performs sampling or optimization in the latent space to generate molecules.
- Objective-Reinforced Generative Adversarial Networks (ORGAN)^15^ is a reinforcement learning based molecule generation algorithm that uses SMILES strings for input and output.
- Graph Convolutional Policy Network (GCPN)^18^ is another reinforcement learning based algorithm that operates on a graph representation of molecules in combination with a MDP.

### Single property optimization

In this task, our goal is to find a molecule that can maximize one selected property. Similar to the setup in previous approaches^13,18^, we demonstrated the property optimization task on two targets: penalized logP and Quantitative Estimate of Druglikeness (QED)^28^. LogP is the logarithm of the partition ratio of the solute between octanol and water. Penalized logP^13^ is the logP minus the synthetic accessibility (SA) score and the number of long cycles.

**Figure 2:**
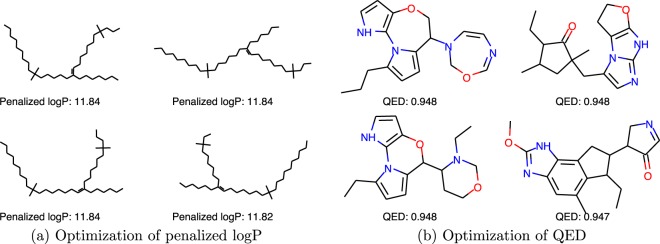
Sample molecules in the property optimization task. (**a**) Optimization of penalized logP from MolDQN-bootstrap; note that the generated molecules are obviously not drug-like due to the use of a single-objective reward. (**b**) Optimization of QED from MolDQN-twosteps.

In this experiment setup, the reward was set to be the penalized logP or QED score of the molecule. For logP optimization, the initial molecule was set to be empty, while for QED optimization, a two-step optimization was used to improve the result. The first step started with an empty molecule, and the second step started with the 5 molecules that have the highest QED values found in step one. The max number of steps per episode for LogP optimization is set to be 38, in order to allow a direct comparison with GCPN. We will discuss the rationale for this choice in later paragraphs. This number is set to 40 in QED optimization. We picked the last 100 terminal states in the training process and report the top three property scores found by each model and the percentage of valid molecules in Table 1. Note that the range of penalized logP is $(\,-\,\infty ,\infty )$, while the range of QED is $[0,1]$. We also visualized the best molecules we found in Fig. 2. Note that in the optimization of penalized logP, the generated molecules are obviously not drug-like, which highlights the importance of carefully designing the reward (including using multiple objectives in a medicinal chemistry setting) when using reinforcement learning.

**Table 1:** Top three unique molecule property scores found by each method.

	Penalized logP				QED			
	1st	2nd	3rd	Validity	1st	2nd	3rd	Validity
random walk^a^	−3.99	−4.31	−4.37	100%	0.64	0.56	0.56	100%
greedy^b^	11.41	—	—	100%	0.39	—	—	100%
$\varepsilon$-greedy, $\varepsilon =0.1$^b^	11.64	11.40	11.40	100%	0.914	0.910	0.906	100%
JT-VAE^c^	5.30	4.93	4.49	100%	0.925	0.911	0.910	100%
ORGAN^c^	3.63	3.49	3.44	0.4%	0.896	0.824	0.820	2.2%
GCPN^c^	7.98	7.85	7.80	100%	0.948	0.947	0.946	100%
MolDQN-naïve	11.51	11.51	11.50	100%	0.934	0.931	0.930	100%
MolDQN-bootstrap	11.84	11.84	11.82	100%	0.948	0.944	0.943	100%
MolDQN-twosteps	—	—	—	—	0.948	0.948	0.948	100%

^a^“random walk” is a baseline that chooses a random action for each step.

^b^“greedy” is a baseline that chooses the action that leads to the molecule with the highest reward for each step. “$\varepsilon$-greedy” follows the “random” policy with probability $\varepsilon$, and “greedy” policy with probability $1-\varepsilon$. In contrast, the $\varepsilon$-greedy MolDQN models choose actions based on predicted *Q*-values rather than rewards.

^c^values are reported in You *et al*.^18^.

We compared our model to three baselines. “Random walk” is a baseline that chooses a random action for each step, “greedy” is a baseline that chooses the action that leads to the molecule with the highest reward for each step, and “$\varepsilon$-greedy” follows the “random” policy with probability $\varepsilon$, and “greedy” policy with probability $1-\varepsilon$. Additionally, we compared our model to three published literature models: ORGAN^15^, JT-VAE^13^, and GCPN^18^.

With the introduction of bootstrapped DQN, we are able to find molecules with higher QED values compared to naive DQN, demonstrating the exploration efficiency of bootstrapping. However, on the task of maximizing penalized logP, bootstrapped DQN does not provide a significantly better result. This is partly because maximizing logP corresponds to a simple policy: adding carbon atoms wherever possible. This straightforward policy does not require much exploration effort, and can be regarded as a greedy policy (Table 1).

Moreover, our experiments reveal that the task of maximizing logP with no constraints is not a good metric to evaluate the performance of a model. The penalized logP value almost increases linearly with the number of atoms (Fig. S7), therefore it is not fair to compare logP without limiting the number of atoms to be the same. Although the task of optimizing logP can be used to evaluate whether a model can capture the simple domain-specific heuristic, we suggest that maximization should be performed under certain constraints, for example, number of atoms, or similarity. We also suggest that targeting a specific range of logP is also a valid task to evaluate the performance of different models. This task not only avoids the problem of unconstrained optimization, but also represents a real need in typical drug discovery projects.

Compared with GCPN, MolDQN demonstrates better performance on the task of logP, and similar performance on the task of QED. These results can be partly attributed to learning from scratch, where the scope is not limited to the molecules in a specific dataset.

Note that we can also start from an existing molecule for optimization. In Section S1.1, we demonstrate optimizations starting from 30 different molecules in ChEMBL for two different target synthetic accessibility (SA) scores.

### Constrained optimization

We performed molecule optimization under a specific constraint, where the goal is to find a molecule *m* that has the largest improvement compared to the original molecule *m*_0_, while maintaining similarity ${\rm{SIM}}(m,{m}_{0})\ge \delta$ for a threshold *δ*. Here we defined the similarity as the Tanimoto similarity^a^^1^ between Morgan fingerprints^26^ with radius 2 of the generated molecule *m* and the original molecule *m*_0_. Following the experiment in Jin *et al*.^13^, we trained a model in an environment whose initial state was randomly set to be one of the 800 molecules in ZINC^29^ dataset which have the *lowest* penalized logP value, and ran the trained model on each molecule for one episode. The maximum number of steps per episode was limited to 20 in consideration of computational efficiency. In this task, the reward was designed as follows:

$$ {\mathcal R} (s)=\{\begin{array}{ll}{\rm{logP}}(m)-\lambda \times (\delta -{\rm{SIM}}(m,{m}_{0})) & {\rm{if}}\,{\rm{SIM}}(m,{m}_{0}) < \delta \\ {\rm{logP}}(m) & {\rm{otherwise}}\end{array}$$

where *λ* is the coefficient to balance the similarity and logP. If the similarity constraint is not satisfied, the reward is penalized by the difference between the target and current similarity. In our experiments $\lambda =100$. We report the success rate—the percentage of molecules satisfying the similarity constraint—as validity, as well as the average improvement on logP in Table 2. Using Welch’s *t*-test^30^ for $N=800$ molecules, we found that both variants of MolDQN gives a highly statistically significant improvement over GCPN for all values of *δ* with $t < -\,8$. The bootstrap variant also significantly outperforms the naive model (except for $\delta =0.2$) with $t < -\,3$.

**Table 2:** Mean and standard deviation of penalized logP improvement in constraint optimization tasks.

*δ*	JT-VAE^a^		GCPN^a^		MolDQN-naive		MolDQN-bootstrap	
	Improvement	Success	Improvement	Success	Improvement	Success	Improvement	Success
0.0	1.91 ± 2.04	97.5%	4.20 ± 1.28	100%	6.83 ± 1.30	100%	7.04 ± 1.42	100%
0.2	1.68 ± 1.85	97.1%	4.12 ± 1.19	100%	5.00 ± 1.55	100%	5.06 ± 1.79	100%
0.4	0.84 ± 1.45	83.6%	2.49 ± 1.30	100%	3.13 ± 1.57	100%	3.37 ± 1.62	100%
0.6	0.21 ± 0.71	46.4%	0.79 ± 0.63	100%	1.40 ± 1.05	100%	1.86 ± 1.21	100%

*δ* is the threshold of the similarity constraint ${\rm{SIM}}(m,{m}_{0})\ge \delta$. The success rate is the percentage of molecules satisfying the similarity constraint.

^a^values are reported in You *et al*.^18^.

### Multi-objective optimization

In drug design, there is often a minimal structural basis that a molecule must retain to bind a specific target, referred to as the molecular scaffold. This scaffold is usually defined as a molecule with removal of all side chain atoms^31^. Often the question arises: can we find a molecule similar to a existing one but having a better performance? We designed the experiment of maximizing the QED of a molecule while keeping it similar to a starting molecule. The multi-objective reward of a molecule *m* was set to be a 2-dimensional vector of $\overrightarrow{r}=[{\rm{QED}}(m),{\rm{SIM}}(m,{m}_{0})]$, where ${\rm{QED}}(m)$ is the QED score and ${\rm{SIM}}(m,{m}_{0})$ is the Tanimoto similarity between the Morgan fingerprints of molecule *m* and the original molecule *m*_0_.

Different weights *w* can be applied to denote the priorities of these two objectives. The variable *w* denotes the weight of similarity score, while the QED score is balanced by $(1-w)$. This is referred to as a “scalarized” multi-objective optimization strategy (see Section 2.3):

$$ {\mathcal R} (s)=w\times {\rm{SIM}}(s)+(1-w)\times {\rm{QED}}(s)$$

**Figure 3:**
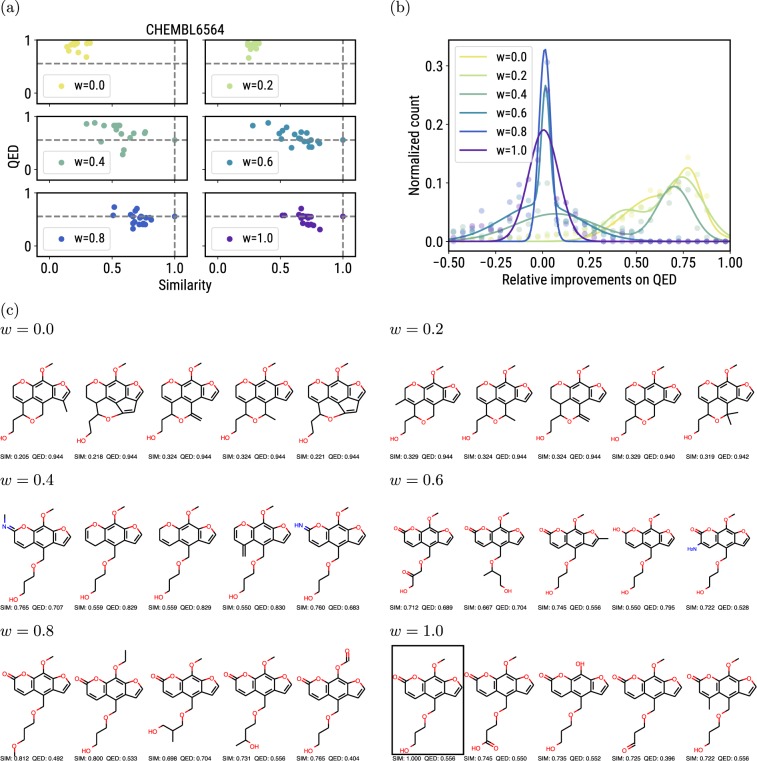
(**a**) The QED and Tanimoto similarity of the molecules optimized under different objective weights. The grey dashed line shows the QED and similarity score of the starting molecule. The legends are transparent, thus it will not cover any point. (**b**) The empirical distribution of the relative QED improvements in 20 multi-objective optimization tasks. The variable *w* in legends denotes the weight of the similarity in the multi-objective reward, while the QED score is weighted by $(1-w)$, i.e. $r=w\times {\rm{SIM}}(s)+(1-w)\times {\rm{QED}}(s)$. (**c**) Unique molecules sampled from the multi-objective optimization task. The original molecule is boxed.

We trained the model with objective weight of 0.0, 0.2, 0.4, 0.6, 0.8, and 1.0, and collected the last 20 unique molecules generated in the training process to plot the properties of molecules on a 2-D space. (i.e., there xwas no separate evaluation step). Figure 3a shows the properties of the optimized molecules under different weights. Figure 3a demonstrates that we can successfully optimize the QED of a molecule while keeping the optimized molecule similar to the starting molecule. As the weight applied on similarity increases, the optimized molecules have higher similarity to the starting molecule, and larger fractions of the optimized molecules have QED values lower than those of the starting molecules. The same experiment was repeated for 20 molecules randomly selected from ChEMBL^32^ (Fig. S1), and the empirical distribution of the relative improvement of QED was plotted in Fig. 3b, where the relative improvement of molecule *m* with respect to the original molecule *m*_0_ is defined as

$${{\rm{imp}}}_{rel}=\frac{{\rm{QED}}(m)-{\rm{QED}}({m}_{0})}{1-{\rm{QED}}({m}_{0})}$$

Intuitively, the relative improvement is the ratio of the actual improvement to the largest possible improvement in QED. The distribution of absolute QED improvement is shown in Fig. S6.

As the weight on similarity increases, the distribution of QED improvements moves leftwards because higher priority is placed on similarity. Finally, we visually examined the optimized molecules (Fig. 3c). The molecules generated under *w* >= 0.4 possessed the same scaffold as the starting molecule, indicating that the trained model preserves the original scaffold when the similarity weight is high enough.

### Optimality vs. diversity

Related work in this area reports results for two distinct tasks: optimization and generation (or, to avoid ambiguity, *property-directed sampling*). Optimization is the task to find the best molecule with regard to some objectives, whereas property-directed sampling is the task of generating a set of molecules with specific property values or distributions.

For the results we report in this paper, we note that there is often a trade-off between optimality and diversity. Without the introduction of randomness, execution of our learned policy will lead to *exactly one* molecule. Alternatively, there are three possible ways to increase the diversity of the molecules generated:

1. Choose one *Q* function ${Q}^{(i)}(s,a)$ uniformly for *i* in $1,\ldots ,H$ to make decision in each episode.
2. Draw an action stochastically with probability proportional to the *Q*-function in each step (as in Haarnoja *et al*.^33^).
3. During evaluation, use non-zero $\varepsilon$ in the $\varepsilon$-greedy algorithm.

In Strategy 1, we are following the action maximizing the *Q*-function, which is an optimal choice. However, this strategy is slightly less optimal than using a single *Q*-function in the sense that each *Q*-function is only trained on a subset of the samples. Strategies 2 and 3 are clearly sub-optimal because the policy is no longer pursuing the maximum future rewards. In the results above, we focused primarily on optimization tasks and leave the question of diversity for future work.

We also conducted experiments to illustrate that we are able to find molecules with properties in specific ranges with 100% success (Table S1). In addition, we demonstrated that we can generate molecules that satisfy multiple target values (Table S2). However, because we formulated the property targeting to be an optimization task, it is not fair for us to compare to other generative models that produce diverse distributions of molecules.

### Visualization and Interpretation

Users prefer interpretable solutions when they applying methods that construct new molecules. Here we demonstrated the decision making process of MolDQN that maximizes the QED, starting from a specific molecule.

**Figure 4:**
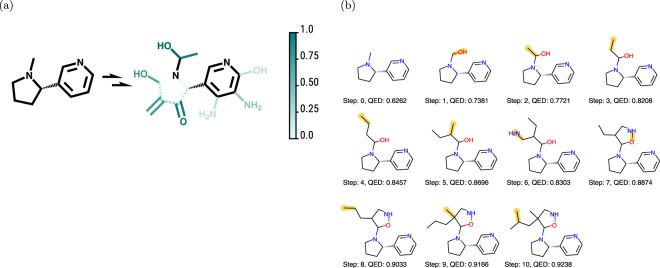
(**a**) Visualization of the *Q*-values of selected actions. The full set of *Q*-values of actions are shown in Fig. S2. The original atoms and bonds are shown in black while modified ones are colored. Dashed lines denote bond removals. The *Q*-values are rescaled to $[0,1]$. (**b**) The steps taken to maximize the QED starting from a molecule. The modifications are highlighted in yellow. The QED values are presented under the modified molecules.

In the first step of decision making, the *Q*-network predicts the *Q*-value of each action. Figure 4a shows the predicted *Q*-values of the chosen actions. The full set of *Q*-values of for all actions in the first step are shown in Fig. S2. We observe that adding a hydroxyl group is strongly favored, while breaking the five-member ring structure is disfavored.

Note that the *Q*-value is a measure of future rewards; therefore, it is possible for the algorithm to choose an action that decreases the property value in the short term but can reach higher future rewards. Figure 4b shows a sample trajectory of maximizing the QED of a molecule (note that $\varepsilon =0$ during evaluation). In this trajectory, step 6 decreases the QED of the molecule, but the QED was improved by 0.297 through the whole trajectory.

## Conclusion

By combining state-of-the-art deep reinforcement learning with domain knowledge of chemistry, we developed the MolDQN model for molecule optimization. We demonstrated that MolDQN reaches equivalent or better performance when compared with several other established algorithms in generating molecules with better specified properties. We also presented a way to visualize the decision making process to facilitate learning a strategy for optimizing molecular design. Future work can be done on applying different *Q*-function approximators (for example MPNN^34^) and hyperparameter searching. We hope the MolDQN model will assist medicinal and material chemists in molecular design.

As a parting note, it seems obvious to us that the experiments and metrics commonly employed in the literature (including this work) are inadequate for evaluating and comparing generative models in real-world optimization tasks. In particular, logP is a “broken” metric that should be discouraged except as a sanity check, and many other commonly used metrics such as QED suffer from boundary effects that limit comparability. Additionally, “computable” metrics like QED should be deprioritized in favor of therapeutically relevant properties that can be verified by experiment—this likely requires incorporating predictive models based on experiment into generative decision making, as in Li *et al*.^11^. Even better would be to couple these predictions with experimental validation, as has been done by Merk *et al*.^35^ and Putin *et al*.^6^. We note that some efforts have been made in addressing generator evaluation^36^, but there remains much work to be done to fairly compare one model to another on meaningful tasks and make these models relevant and effective in prospective drug discovery.

## Supplementary information

Supporting Information for Optimization of Molecules via Deep Reinforcement Learning

## Data Availability

The ChEMBL^32^ and ZINC^29^ datasets used in this study are available online. No dataset was generated during the current study. The code is available at https://github.com/google-research/google-research/tree/master/mol_dqn.

## Footnotes

1 The Tanimoto similarity uses the ratio of the intersecting set to the union set as the measure of similarity. Represented as a mathematical equation $T(a,b)=\tfrac{{N}_{c}}{{N}_{a}+{N}_{b}-{N}_{c}}$. *N*_*a*_ and *N*_*b*_ represents the number of attributes in each object (*a*, *b*). *N*_*c*_ is the number of attributes in common.
