# Author Correction: Optimization of Molecules via Deep Reinforcement Learning

**DOI:** 10.1038/s41598-020-66840-x

**Published:** 2020-06-23

**Authors:** Zhenpeng Zhou, Steven Kearnes, Li Li, Richard N. Zare, Patrick Riley

**Affiliations:** 1 Department of Chemistry, Stanford University, Stanford, California USA; 2 Google Research Applied Science, Mountain View, California USA; 3 Work done during an internship at Google Research Applied Science, Mountain View, California USA

Correction to: *Scientific Reports* 10.1038/s41598-019-47148-x, published online 24 July 2019

This Article contains errors.

Penalized logP is reported in this Article for our methods in a non-normalised form. Previous research (which we compared to in the original Tables 1 and 2), starting with JT-VAE^1^ normalized the logP to be zero mean and unit standard deviation based on the training set. To correctly compare with previous research, our penalized logP was recalculated into a normalised form. This resulted in changes of numerical values for Penalized logP in Table 1, and numerical values for MolDQN-naïve and MolDQN-bootstrap Improvement in Table 2. The corrected versions of these tables are included below as Tables 1 and 2.

**Table 1:** Top three unique molecule property scores found by each method.

	Penalized logP				QED			
	1st	2nd	3rd	Validity	1st	2nd	3rd	Validity
random walk^a^	−0.65	−1.72	−1.88	100%	0.64	0.56	0.56	100%
greedy^b^	9.05	—	—	100%	0.39	—	—	100%
ε-greedy, ε = 0.1^b^	9.10	9.05	9.05	100%	0.914	0.910	0.906	100%
JT-VAE^c^	5.30	4.93	4.49	100%	0.925	0.911	0.910	100%
ORGAN^c^	3.63	3.49	3.44	0.4%	0.896	0.824	0.820	2.2%
GCPN^c^	7.98	7.85	7.80	100%	0.948	0.947	0.946	100%
MolDQN-naive	8.69	8.68	8.67	100%	0.934	0.931	0.930	100%
MolDQN-bootstrap	9.01	9.01	8.99	100%	0.948	0.944	0.943	100%
MolDQN-bootstrap	—	—	—	—	0.948	0.948	0.948	100%

^a^“random walk” is a baseline that chooses a random action for each step.

^b^“greedy” is a baseline that chooses the action that leads to the molecule with the highest reward for each step. “ε-greedy” follows the “random” policy with probability ε, and “greedy” policy with probability 1–ε. In contrast, the ε-greedy MolDQN models choose actions based on predicted Q-values rather than rewards.

^c^values are reported in You *et al*.^2^.

**Table 2:** Mean and standard deviation of penalized logP improvement in constrained optimization tasks.

δ	JT-VAE^a^		GCPN^a^		MolDQN-naive		MolDQN-bootstrap	
	Improvement	Success	Improvement	Success	Improvement	Success	Improvement	Success
0.0	1.91 ± 2.04	97.5%	4.20 ± 1.28	100%	4.83 ± 1.30	100%	4.88 ± 1.30	100%
0.2	1.68 ± 1.85	97.1%	4.12 ± 1.19	100%	3.79 ± 1.32	100%	3.80 ± 1.30	100%
0.4	0.84 ± 1.45	83.6%	2.49 ± 1.30	100%	2.34 ± 1.18	100%	2.44 ± 1.25	100%
0.6	0.21 ± 0.71	46.4%	0.79 ± 0.63	100%	1.40 ± 0.92	100%	1.30 ± 0.98	100%

δ is the threshold of the similarity constraint SIM(m,m_0_) ≥ δ. The success rate is the percentage of molecules satisfying the similarity constraint.

^a^values are reported in You *et al*.^2^.

Additionally, in the Discussion, subsection “Constrained optimization”:

“Using Welch’s *t*-test^30^ for *N* = 800 molecules, we found that both variants of MolDQN gives a highly statistically significant improvement over GCPN for all values of *δ* with *t* < −8. The bootstrap variant also significantly outperforms the naive model (except for *δ* = 0.2) with *t* < −3.”

should read:

“Using Welch’s *t*-test^30^, we found that on *δ* = 0.2, both variants of MolDQN gives a statistically significant lower improvement comparing to GCPN with *P* < 1e-7; on *δ* = 0.4, the differences are insignificant at a 1% level with *P* = 0.016 for MolDQN naive and *P* = 0.43 for MolDQN bootstrap; on *δ* = 0.0 and 0.6, both variants of MolDQN gives a statistically significant higher improvement comparing to GCPN with *P* < 1e-22. The differences between two variants of MolDQN are statistically insignificant at a 1% level with *P* > 0.036.”

Furthermore, Figure S7 was updated. The corrected version is shown below as Figure 1.

**Figure 1:**
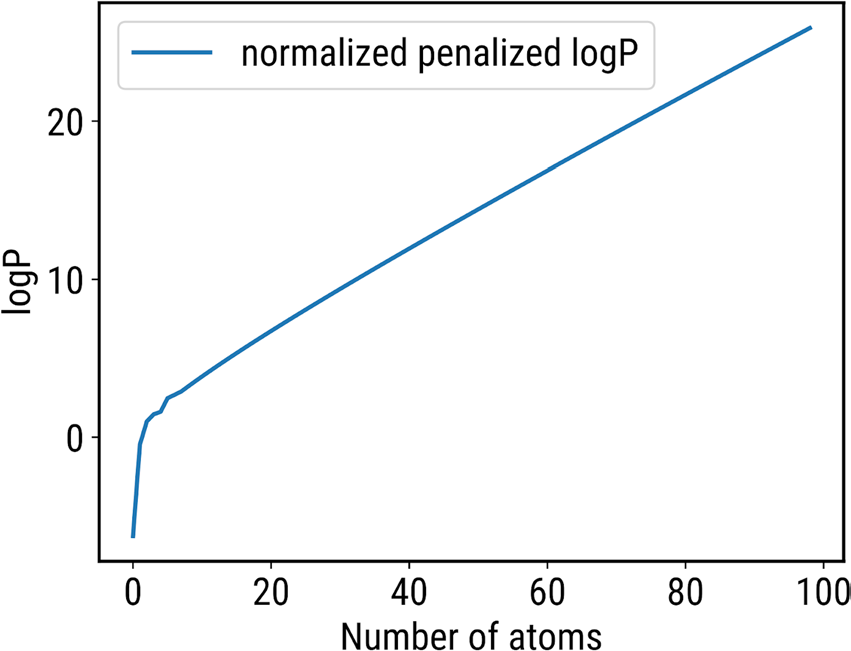
.

Lastly, References 13 and 18 were incorrectly given as:

13. Jin, W., Barzilay, R. & Jaakkola, T. Junction tree variational autoencoder for molecular graph generation. arXiv preprint arXiv:1802.04364 (2018).

18. You, J., Liu, B., Ying, R., Pande, V. & Leskovec, J. Graph convolutional policy network for goal-directed molecular graph generation. arXiv preprint arXiv:1806.02473 (2018).

The correct references are listed below as references 1 and 2.

These changes do not affect the conclusions of the Article.
