# Supplementary material for: Optimization of Molecules via Deep Reinforcement Learning
Source: Sci Rep. 2019 Jul 24;9:10752. doi: 10.1038/s41598-019-47148-x (PMC6656766; doi:10.1038/s41598-019-47148-x)
Supplement: Supplementary file 1 — Supporting Information for Optimization of Molecules via Deep Reinforcement Learning [file 41598_2019_47148_MOESM1_ESM.pdf]

Supporting Information for Optimization of Molecules via Deep Reinforcement Learning

Zhenpeng Zhou,[*,†,¶] Steven Kearnes,[*,‡] Li Li,[*,‡] Richard N. Zare,[*,†] and Patrick Riley[*,‡]

†*Department of Chemistry, Stanford University*

‡*Google AI Applied Science*

¶*Work done during an internship at Google AI Applied Science*

E-mail: zhenpeng@stanford.edu; kearnes@google.com; leeley@google.com; rnz@stanford.edu; pfr@google.com

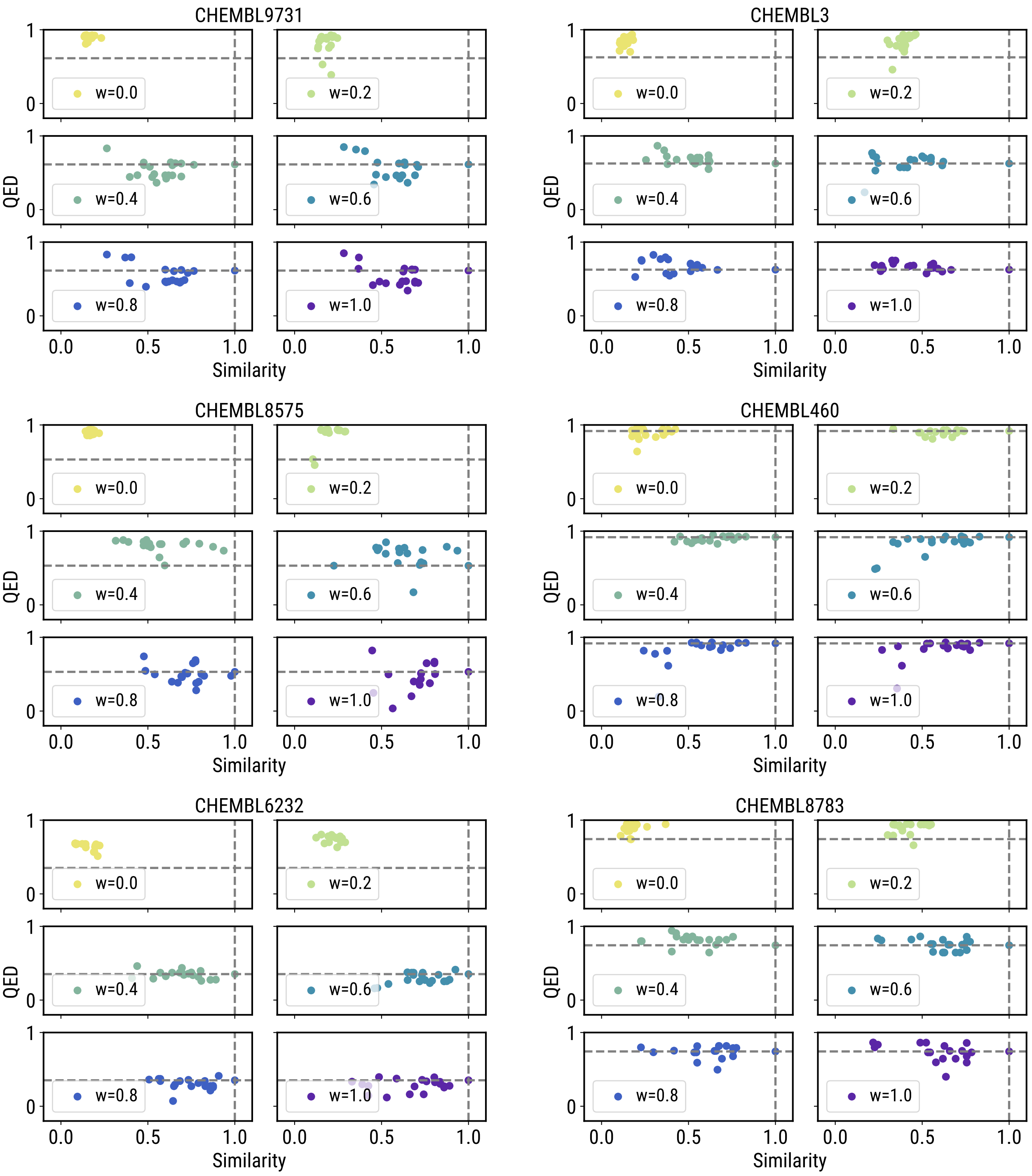
CHEMBL9731
CHEMBL3
CHEMBL8575
CHEMBL460
CHEMBL6232
CHEMBL8783
QED
Similarity
w=0.0
w=0.2
w=0.4
w=0.6
w=0.8
w=1.0
0
1
0.0
0.5
1.0

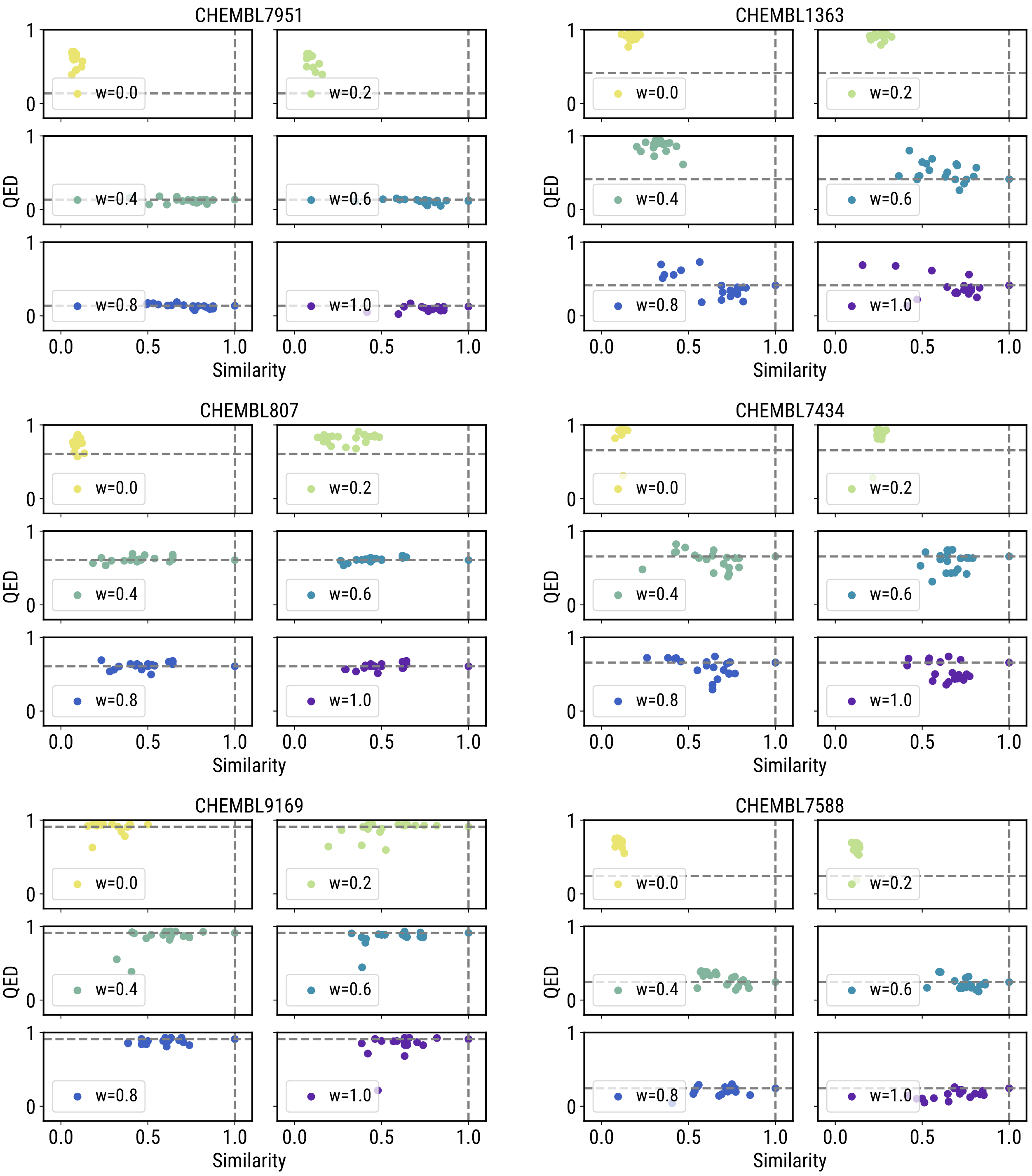

CHEMBL7951
CHEMBL1363
CHEMBL807
CHEMBL7434
CHEMBL9169
CHEMBL7588
QED
Similarity
w=0.0
w=0.2
w=0.4
w=0.6
w=0.8
w=1.0
0
1
0.0
0.5
1.0

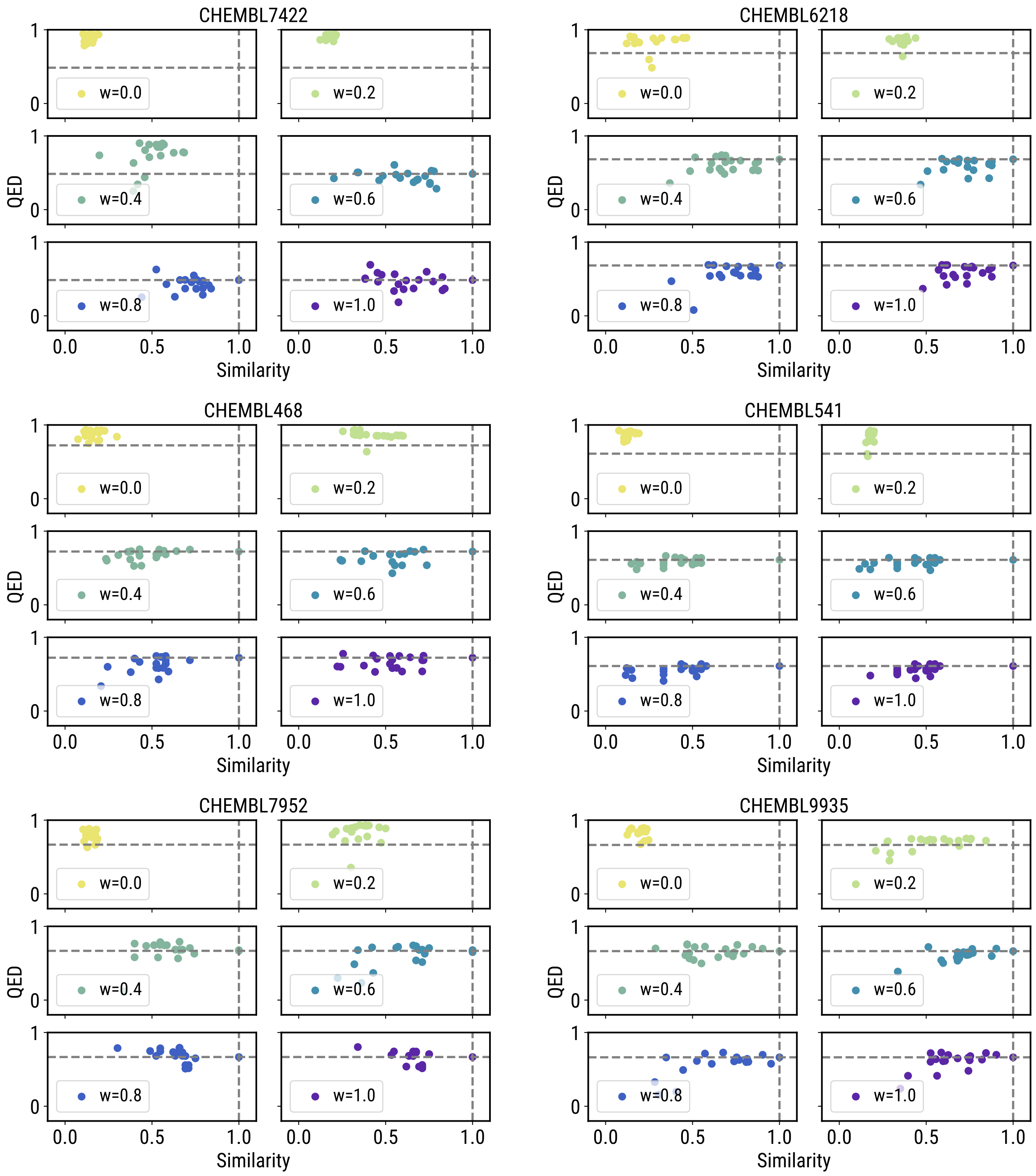
CHEMBL7422
CHEMBL6218
CHEMBL468
CHEMBL541
CHEMBL7952
CHEMBL9935
QED
Similarity
w=0.0
w=0.2
w=0.4
w=0.6
w=0.8
w=1.0

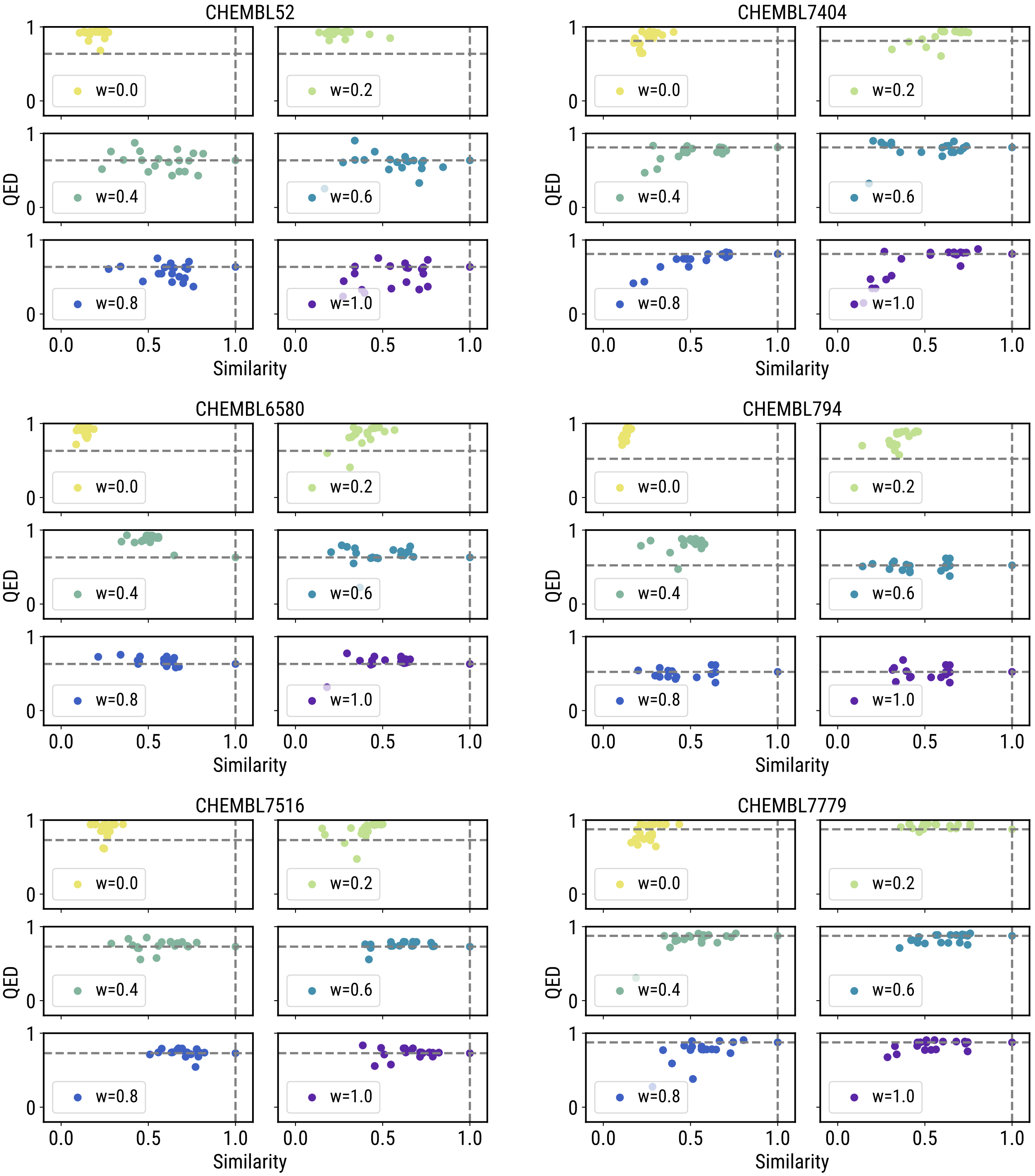

Figure S1: The QED and similarity of the molecules generated under different weights with different staring molecules. The gray dash line shows the QED and similarity score of the starting molecule.

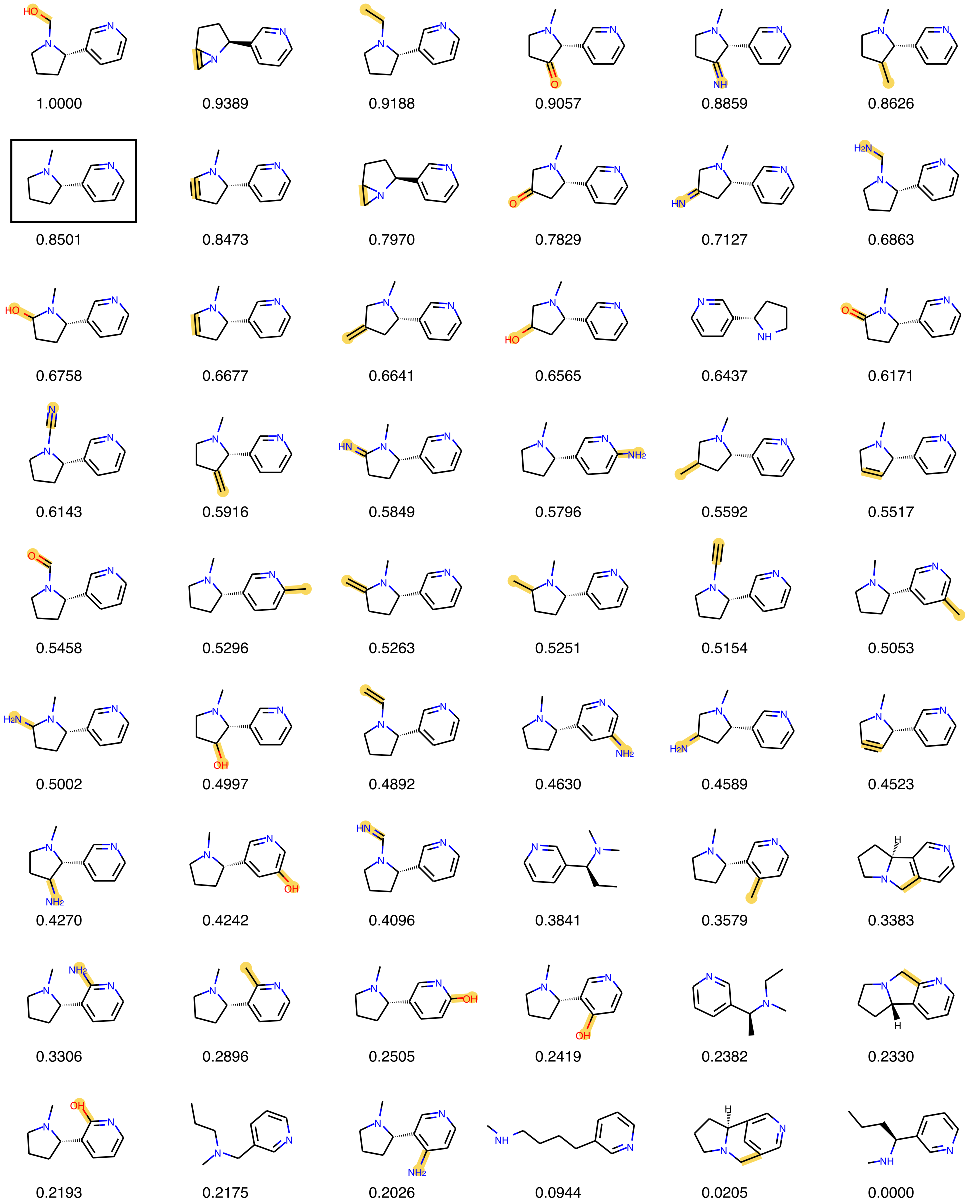

Figure S2: The normalized Q-values of the actions can be taken in the first step. The original molecule was boxed. Bond addition actions are highlighted while bond removals are presented as is. The Q-values are rescaled to $[0, 1]$

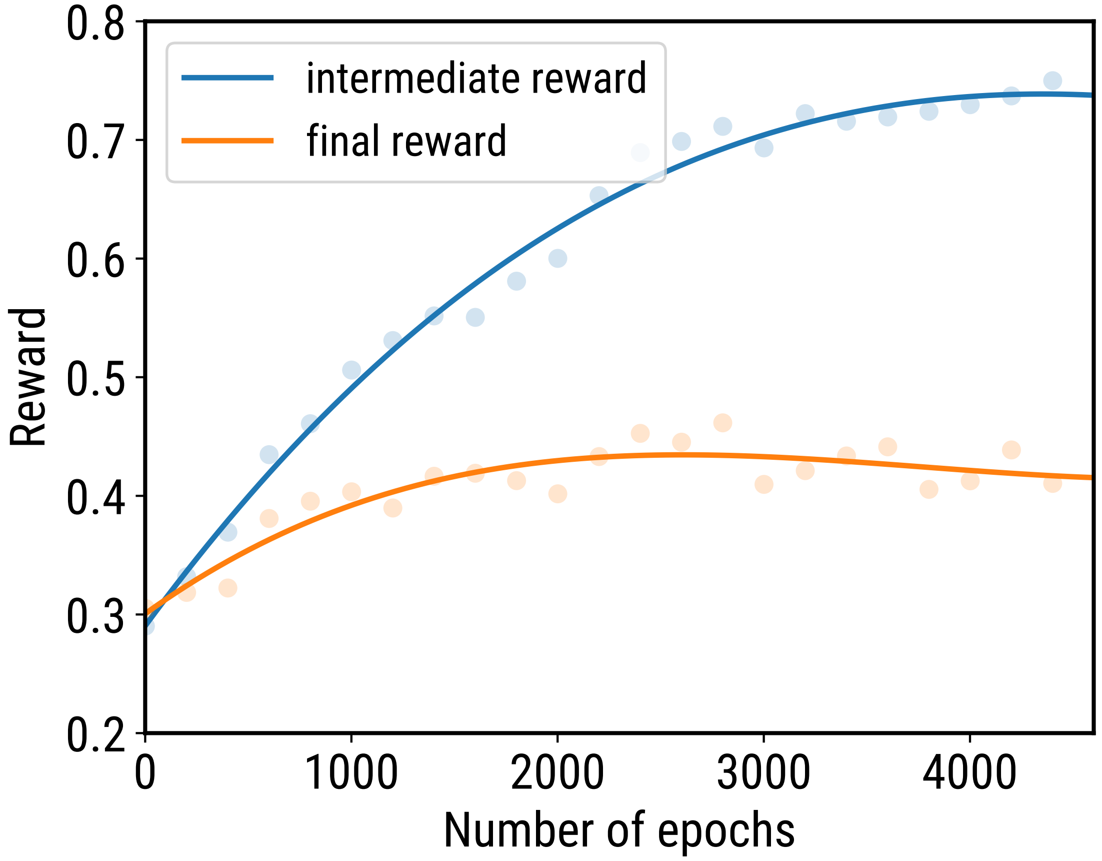

Figure S3: Comparison between the learning curve of the agent while intermediate reward is given and that when only final reward is given. Here reward is defined as the QED of the final molecule generated. Bootstrap is turned off in this experiment.

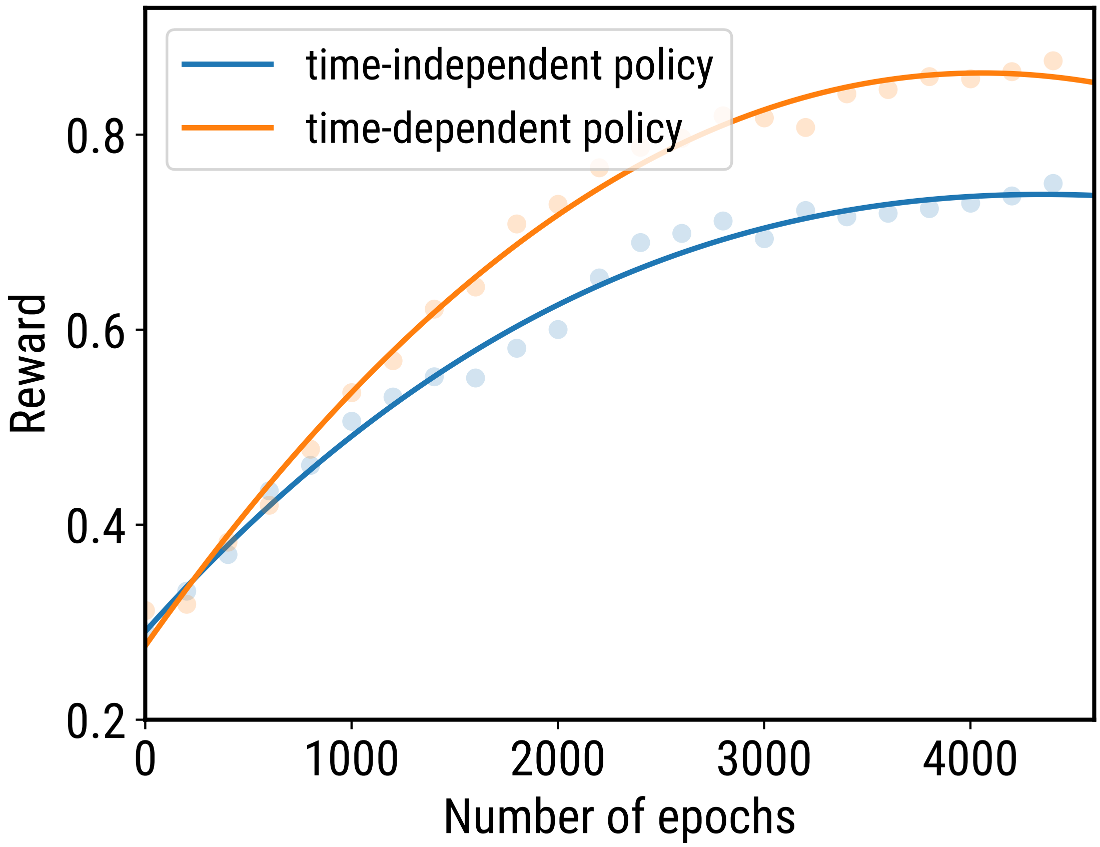

Figure S4: Comparison of a time-dependent policy and a time-independent policy. Here reward is defined as the QED of the final molecule generated. Bootstrap is turned off in this experiment.

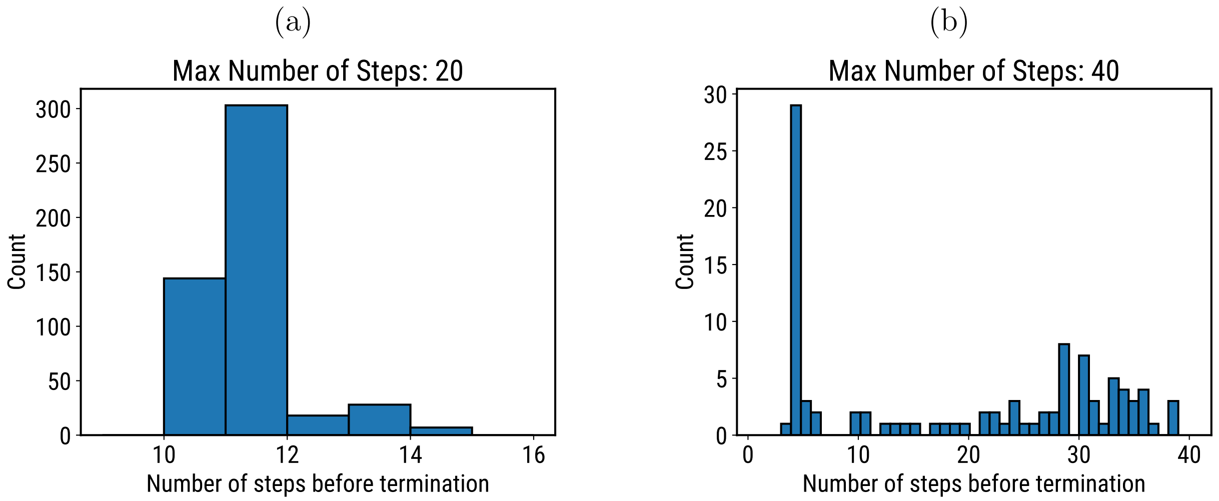

Figure S5: Histogram of number of steps before the policy chooses to stay at the same step (the "no modification" action, all subsequent actions are "no modification"). (a)The task is to find a molecule whose molecular weight lies between 150 and 200. (b) The task is to find a molecule that maximizes the QED. Bootstrap is turned off in this experiment.

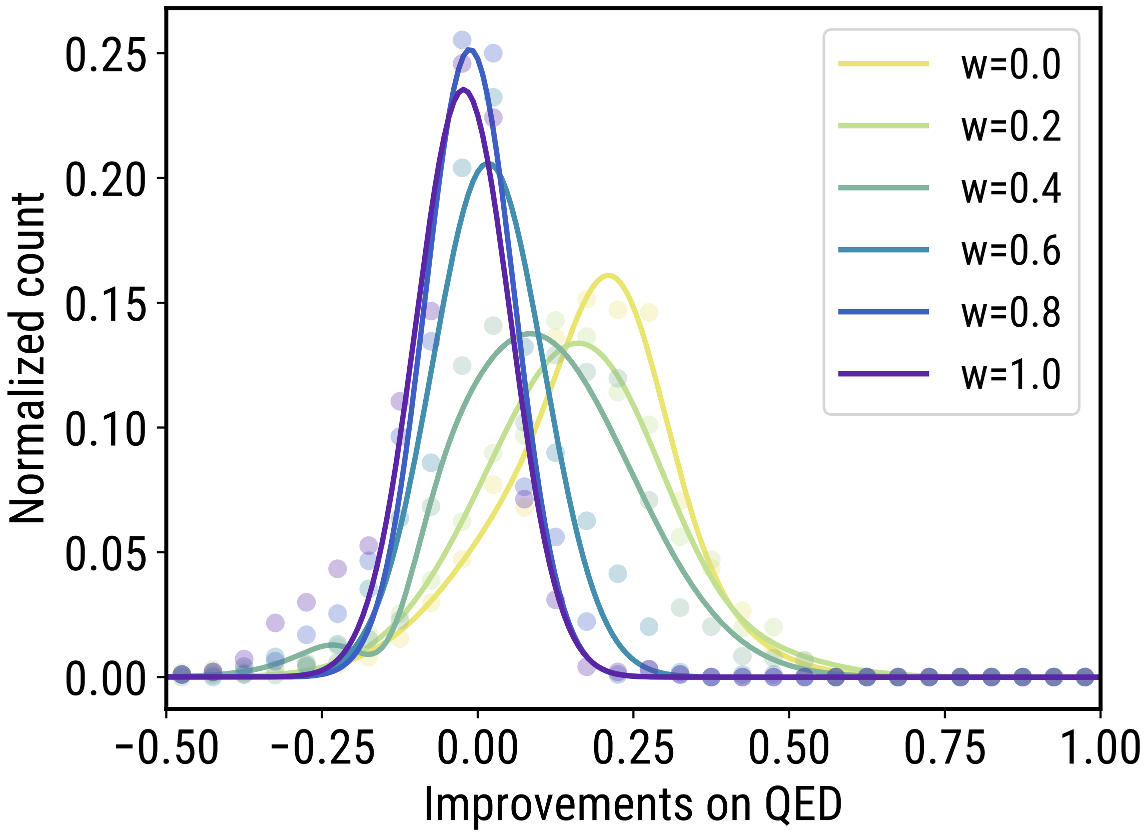

Figure S6: The empirical distribution of the QED improvements in 20 multi-objective optimization tasks. The variable w in legends denotes the weight of the similarity in the multi-objective reward, while the QED score is weighted by $(1-w)$, i.e. $r = w \times \mathrm{SIM}(s) + (1-w) \times \mathrm{QED}(s)$.

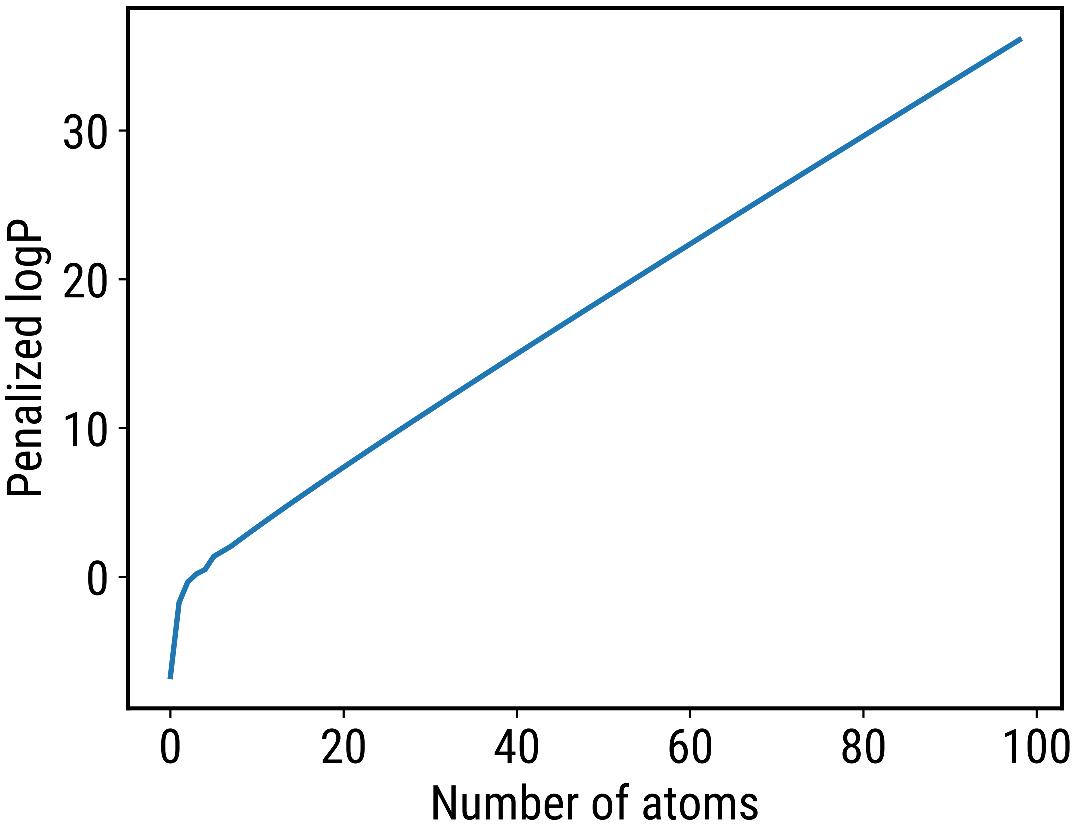

Figure S7: Penalized logP values of acyclic saturated alkane with different number of carbon atoms.

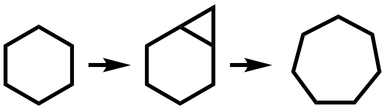

Figure S8: It is still possible to generate a 7-membered ring when only rings with 3 to 6 atoms are allowed.

S1 Property Targeting

S1.1 Single Property Targeting

It is crucial to find molecules with properties close to a given target in molecule design. Molecular weight and hydrophobicity are two important properties in drug design related to drug absorption. Therefore, we chose a target range of molecular weight (MW) and octanol-water partition coefficient (logP), and measured the percentage of the molecules belonging to the specified range. Given a target range of $[l, u]$, where l is the lower bound and u is the upper bound, the reward function of a molecule m is designed as follows:

$$R(s) = \begin{cases} 1 & \text{if} \quad p(m) \in [l, u] \\ -\min\{|p(m) - l|, |p(m) - u|\} & \text{otherwise} \end{cases} \tag{1}$$

where $p(m)$ is the property value of molecule m. Intuitively, this reward is measuring the distance between the current property value and the range we define. When the property value falls into the range we want, a positive reward is given indicating the goal was reached. Otherwise, the reward is the negative minimum distance the property has to "move" in order to be in the desired range. The model was trained using the reward defined in Eq. (1), with the initial molecule being empty. Evaluation was run for 500 episodes, and the properties of the 500 generated molecules are reported.

Table S1: The success rate of the property targeting task.

	-2.5 $\leq$ logP $\leq$ -2	5 $\leq$ logP $\leq$ 5.5	150 $\leq$ Mw $\leq$ 200	500 $\leq$ Mw $\leq$ 550
random walk[a]	0.0%	1.0%	0.0%	0.0%
JT-VAE[b]	11.3%	7.6%	0.7%	16.0%
ORGAN[b]	0.0%	0.2%	15.1%	0.1%
GCPN[b]	85.5%	54.7%	76.1%	74.1%
ours	**100%**	**100%**	**100%**	**100%**

[a] "random walk" is a baseline that chooses a random action for each step.
[b] values are reported in You et al.[1].

Using the same ranges chosen in You et al.[1], the effectiveness of the property targeted

molecule optimization is shown in Table S1. Our model outperforms others by reaching 100% success rates on all tasks. As stated in You et al.[1], the ranges are chosen such that few molecules in ZINC dataset[2] are within that range. Compared with other models, our model do not use expert pretraining on ZINC dataset, therefore the properties of the molecules generated is not limited by the properties of molecules in ZINC.

Note that since our deterministic policy only leads to 1–3 unique molecules, the success rates in Table S1 are not comparable. However, this experiment shows that MolDQN is able to find molecules with arbitrary property values.

We further applied our model on a property targeting task whose objective is to generate molecules with a specific range of synthetic accessibility (SA) score. Comparing with the previous task, we start with drug molecules from the ChEMBL database instead of empty molecules. The reward is defined as:

$$R(s) = R(m, t) = -(\mathrm{SA}(m) - \mathrm{SA}_{\mathrm{target}})^2$$

The model was trained for 5000 epochs with 40 steps per epoch, and evaluated with $\varepsilon = 0$. The SA scores were calculated for the generated molecules at the end of evaluation. The SA scores of the original molecules had a mean of 2.66 and a standard deviation of 0.62. The results are shown in the Figure S9 and Table S2 below:

Table S2: The mean and standard deviation of the SA score of the generated molecules.

target SA score	SA score of the generated molecules
2.5	2.50 ± 0.06
4.8	4.85 ± 0.09

The results show that we can successfully generate molecules with a specific SA score target starting with a drug-like molecule.

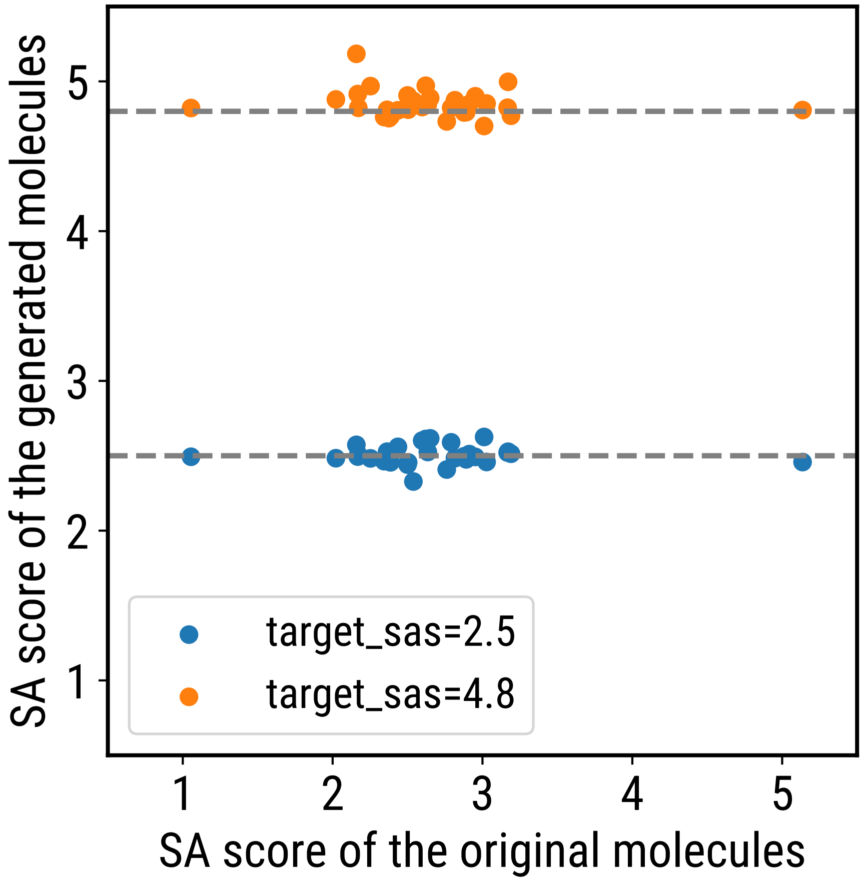

Figure S9: Distributions of the SA score of generated molecules with target scores of 2.5 and 4.8.

S1.2 Multi-Objective Property Targeting

Here we want to illustrate that our model can find optimal molecules that satisfy two constraints at the same time. Similar to the experimental setup in Li et al.[3], the objective is to find optimal molecules which are close to specific Synthetic Accessibility (SA)[4] scores and Quantitative Estimate of Druglikeness (QED)[5] values. Here four different targets are specified as follows: $c_1 = (2.2, 0.84)$, $c_2 = (2.5, 0.27)$, $c_3 = (3.8, 0.84)$, and $c_4 = (4.8, 0.27)$, where the first value is the target SA score and the second is the QED score.

The model was trained using the reward defined below, starting with an empty initial molecule:

$$R(s) = -\left(\left|\text{QED}(m) - \text{QED}_{\text{target}}\right| + \left|\text{SA}(m) - \text{SA}_{\text{target}}\right|\right)$$

Table S3: Property statistics of the optimized molecules.

	Target 1		Target 2		Target 3		Target 4	
	SAS	QED	SAS	QED	SAS	QED	SAS	QED
target value	2.200	0.840	2.500	0.270	3.800	0.840	4.800	0.270
mean [a]	2.303	0.859	2.564	0.251	3.806	0.834	4.799	0.272
standard deviation [a]	0.109	0.012	0.114	0.009	0.074	0.012	0.069	0.005
mean absolute difference [a]	0.103	0.019	0.075	0.020	0.013	0.012	0.009	0.003

[a] Mean, standard deviation, and mean absolute difference denotes the statistics on unique generated molecules.

The distributions of SA score and QED for the molecules generated by our model are shown in Table S3. Even though SA scores and QED may change significantly with small modifications of the molecule, the properties of the generated molecules have a narrow distribution. These results illustrate that explicit rewards on target values can lead to accurate targeted optimization with reinforcement learning.

References

(1) You, J.; Liu, B.; Ying, R.; Pande, V.; Leskovec, J. Graph Convolutional Policy Network for Goal-Directed Molecular Graph Generation. *arXiv preprint arXiv:1806.02473* **2018**,

(2) Irwin, J. J.; Sterling, T.; Mysinger, M. M.; Bolstad, E. S.; Coleman, R. G. ZINC: a free tool to discover chemistry for biology. *Journal of chemical information and modeling* **2012**, *52*, 1757–1768.

(3) Li, Y.; Zhang, L.; Liu, Z. Multi-objective de novo drug design with conditional graph generative model. *Journal of cheminformatics* **2018**, *10*, 33.

(4) Ertl, P.; Schuffenhauer, A. Estimation of synthetic accessibility score of drug-like molecules based on molecular complexity and fragment contributions. *Journal of cheminformatics* **2009**, *1*, 8.

(5) Bickerton, G. R.; Paolini, G. V.; Besnard, J.; Muresan, S.; Hopkins, A. L. Quantifying the chemical beauty of drugs. *Nature chemistry* **2012**, *4*, 90.
